# Older Adults’ Avoidance of Public Transportation after the Outbreak of COVID-19: Korean Subway Evidence

**DOI:** 10.3390/healthcare9040448

**Published:** 2021-04-11

**Authors:** Byungjin Park, Joonmo Cho

**Affiliations:** 1 HRD Center, Department of Economics, Sungkyunkwan University, Seoul 03063, Korea; park0420jin@g.skku.edu; 2 Department of Economics, Sungkyunkwan University, Seoul 03063, Korea

**Keywords:** COVID-19, avoidance of infection, social distancing, free tickets for the aged, subway use demand

## Abstract

With the spread of the coronavirus worldwide, nations have implemented policies restricting the movement of people to minimize the possibility of infection. Although voluntary restriction is a key factor in reducing mobility, it has only been emphasized in terms of the effect of governments’ mobility restriction measures. This research aimed to analyze voluntary mass transportation use after the severe acute respiratory syndrome coronavirus 2 (SARS-CoV-2) outbreak by age group to explore how the perception of the risk of infection affected the public transit system. Mass transportation big data of Seoul Metro transportation use in the capital city of Korea was employed for panel analyses. The analysis results showed that in the period with both the highest and lowest number of infections of SARS-CoV-2, users aged 65 years and over reduced their subway use more than people aged between 20 and 64. This study also found that the decrease in subway use caused by the sharp increase of coronavirus disease 2019 (COVID-19) cases was the most prominent among people aged 65 years and over. The results imply that the elders’ avoidance of public transportation affected their daily lives, consumption, and production activities, as well as their mobility.

## 1. Introduction

Since the World Health Organization (WHO) declared the coronavirus disease 2019 (COVID-19) pandemic, the spread of infections around the world has not abated. While the total number of global COVID-19 cases was 0.7 million in March 2020 when the WHO announced the COVID-19 outbreak a global pandemic, the number of cases exceeded 79.2 million in December 2020 [1,2]. With the incessant outbreaks of community transmission, clusters of cases, and sporadic infections, coronavirus is still spreading throughout the world.

**Figure 1:**
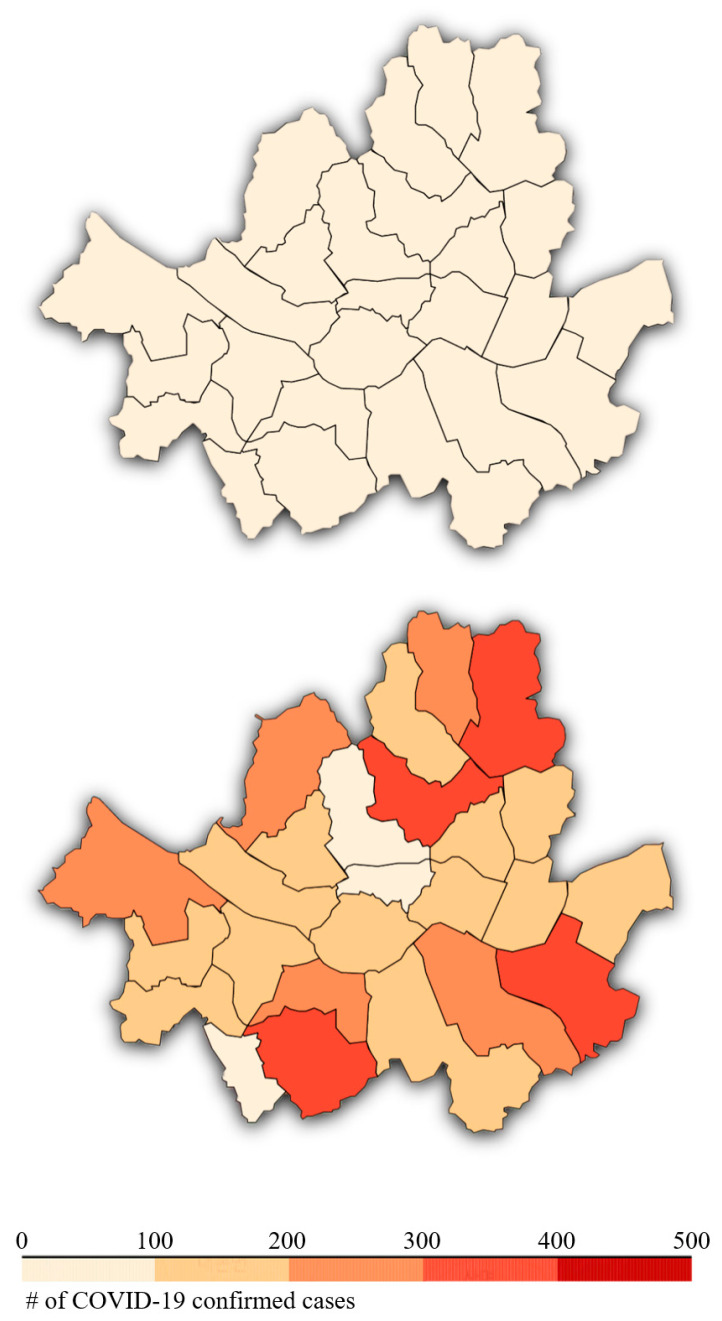
Number of cumulative confirmed cases in Seoul in 2020. The upper and lower figures show the number of infections in February and September, respectively.

The spread of severe acute respiratory syndrome coronavirus 2 (SARS-CoV-2) infections was inevitable, even in Korea. Figure 1 shows the progress of COVID-19 cases in Seoul, the capital of Korea, between February and September. As of December 24, the number of COVID-19 cases in South Korea reached 53,533, with 756 deaths, and the incidence rate (i.e., the cumulative number of confirmed cases per 100,000 population) was 103.25 [3].

With the spread of the coronavirus worldwide, nations implemented policies restricting the movement of people to minimize the amount of contact between people, as WHO reported that COVID-19 is rapidly transmitted through human contact and respiratory organs [4]. Asymptomatic “silent spreaders” were found, making it difficult to track the routes of transmission [5]. Wuhan, the Chinese city where the pandemic is believed to have started, was effectively sealed off from the rest of the country immediately, and movement between regions in China was strictly banned [6]. In Europe, strict restrictions, such as lockdowns, curfews, and permits for movement, were imposed by governments [7]. In the United States, which has the most coronavirus cases worldwide, heightened mobility restrictions, such as stay-at-home orders, bans on gathering, and travel restrictions, were implemented, depending on the status of each state [8].

The Korean government implemented social distancing measures instead of strong restrictions on movement to curb the contagion of SARS-CoV-2. The call for social distancing is a campaign, guidance, or recommendation of the Korean government to achieve regulated mobility through citizens’ voluntary cooperation and compliance. In social distancing phases 1 and 1.5, it was recommended to wear a mask, report meetings and events, and work from home for one-fifth of each institution. In phase 2, all meetings with more than 100 people were prohibited, and a third of employees were encouraged to work from home. Furthermore, the use of entertainment facilities was prohibited and the reduction of store operating hours to 9 p.m. was applied. In phases 2.5 and 3, all of the above restrictions applied to more facilities.

The mobility restriction policies of countries and voluntary restrictions, which were driven by the fear of the coronavirus infection, have led to a decrease in transit demand. In France, consumer mobility, which was estimated based on the use of payment cards, decreased by 75% during the national lockdown compared to 2019 [9]. According to Google Trend data, mass transit demand in ten countries, including Italy, Brazil, and the United Kingdom, fell sharply since the COVID-19 outbreak [10]. In Mexico, the use of automobiles decreased by 10 to 25% since the outbreak [11].

**Figure 2:**
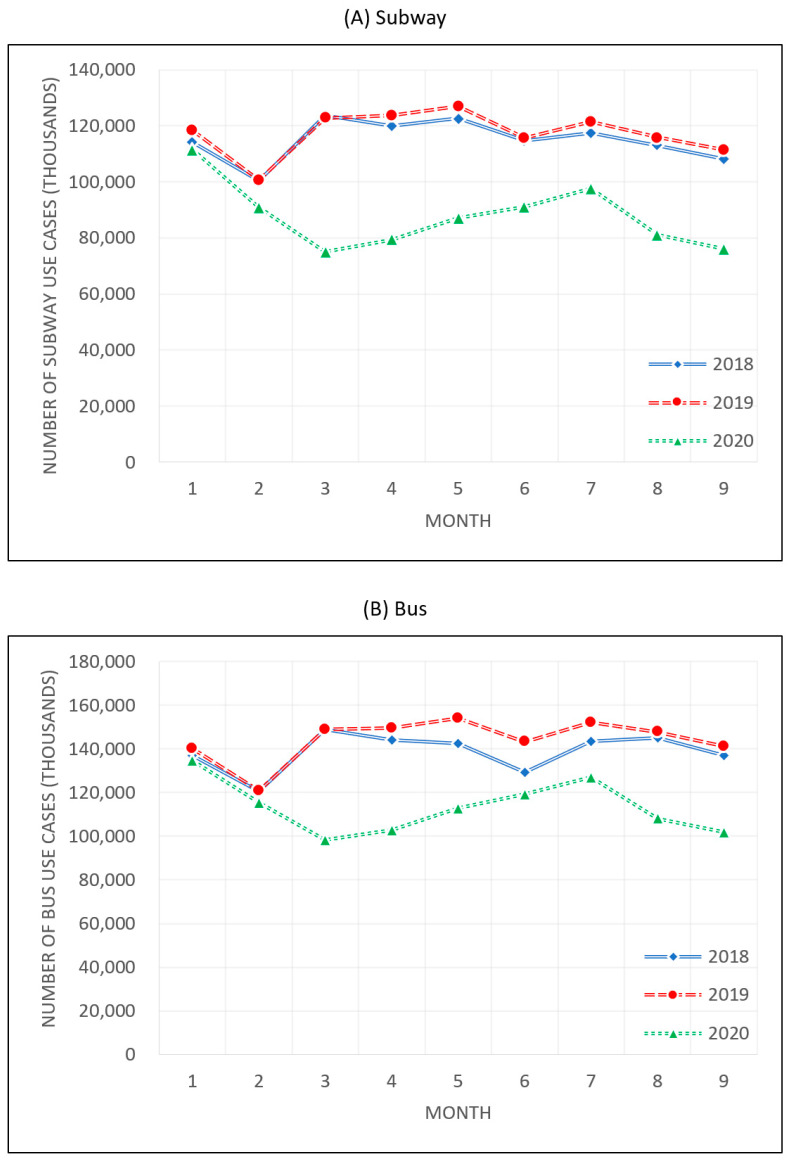
Number of uses of mass transit during 2018–2020.

Seoul has also witnessed a drastic reduction of transit demand amid the pandemic, which can be measured based on the changes in mass transit system use in Seoul, which is among the top ten in the world in terms of efficiency and user satisfaction [12]. The population of Seoul was 9,668,465 as of December 2020, and the number of uses of buses and subways surpassed 100 million each month. Figure 2 shows the number of bus and subway rides in 2018, 2019, and 2020. As the number of cases of COVID-19 began to rise in late January 2020, mass transit demand fell dramatically and rebounded after March, but it was still well below the demand recorded in the same periods of 2018 and 2019. Furthermore, in August, the mass transportation demand declined again after the outbreak of cluster infections in Seoul, showing that mass transportation was significantly affected by the pandemic. Statistics on bus and subway use are in Table A1 of the Appendix A.

Most studies on the decrease of mass transit demand are focused on the effect of governments’ mobility restriction measures. However, there is a lack of studies on the impact of the voluntary restriction that significantly affected the decrease in transit demand. This may be attributed to the fact that, as many countries are implementing restrictions on mobility, it is difficult to isolate the effect of citizens’ voluntary restraint of movement.

Data on subway transit demand in Seoul is useful for studying the voluntary mobility reduction during the pandemic since it has three characteristics that are not found in other cities. First, it is a type of mass transit that carries a high probability of infection. Mass transportation involves very high risks of infection because of the high density, diversity of contacts, and potential presence of patients [13]. Hence, when traveling in the subway, passengers are aware of the risk of infection. Second, the government did not issue any specific restrictions on subway use. Without enforced mobility regulation, subway use reflects the voluntary mobility of people. Third, people aged 65 and over in Seoul can ride a subway for free due to Article 19 of the Welfare of Senior Citizens Act [14]. For the elderly without income earned through economic activity participation, the subway is an essential and the most common means of mass transit for leisure.

However, after the COVID-19 outbreak, subways were classified as facilities that carried the highest infection risk, and the age group most prone to fatality because of a SARS-CoV-2 infection is the elderly group. Therefore, though they have free access to the subway, i.e., the most efficient means of movement, the aged cannot ride on a subway train without concern regarding COVID-19’s impact. As such, the change in the subway ride patterns by the elderly most effectively reflects the perception of the risk of the COVID-19 infection.

The pandemic negatively affected the labor market, as well as people’s perceptions of the risk of infection. At the outbreak of Middle East Respiratory Syndrome, the older adults were the most vulnerable in the labor market, and the confusion in the urban labor market was greater than in the rural areas [15,16]. With the prolonged period of the COVID-19 pandemic, the deterioration of the labor market caused a decline in people’s consumption and income. These changes eventually lead to mobility changes.

This study aimed to empirically analyze the changes in subway use patterns by the elderly and the economically active population amid the risk of SARS-CoV-2 infection. First, the period when cases spiked sharply was separated from other periods for the analysis. The Korean government controlled the social distancing level according to the number of cases. The rise of the social distancing level imposed by the government was intended to heighten citizens’ awareness of the pandemic and cause behavioral change. As such, by focusing on the social distancing level imposed in each period, changes in the behavior of each age group associated with the changes in the social distancing level can be identified. Next, the elasticity of the demand for subway use in response to the number of COVID-19 cases was measured to determine whether the elderly, who are most vulnerable to the coronavirus, were more sensitive to the risk of infection than young people. Lastly, through analysis based on the number of subway stations by period, this study attempted to determine whether changes in subway ride decisions were related to the fear of SARS-CoV-2 infection. In areas near transfer stations (the number of transfer stations was counted based on the number of subway lines available for the station; for instance, for a subway station on three subway lines, the number of stations was counted as three) and multiple stations, there was more transit demand because of easy access to the subway. That is, such areas carry a higher risk of infection from more human contacts. Accordingly, if areas with a greater number of stations are found to experience a higher decrease in passengers than areas with a smaller number of stations, that can be interpreted as a behavioral change to avoid SARS-CoV-2 infection.

### Change in the Pattern of Subway Use Demand Amid the Pandemic in Seoul

**Figure 3:**
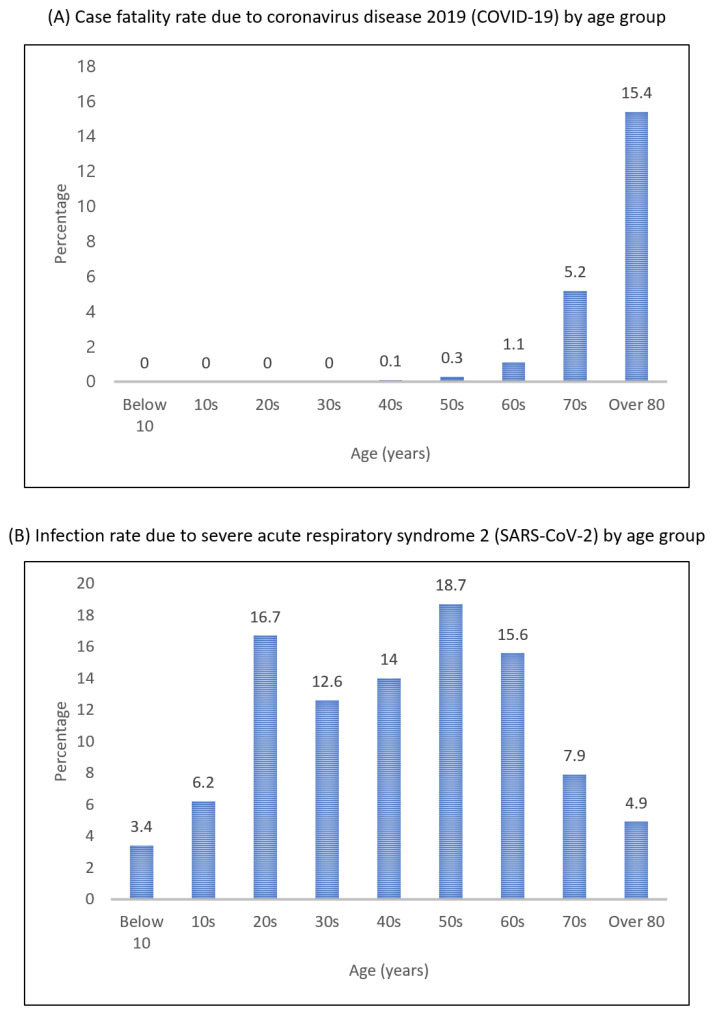
COVID-19 status in South Korea as of 24 December 2020.

**Figure 4:**
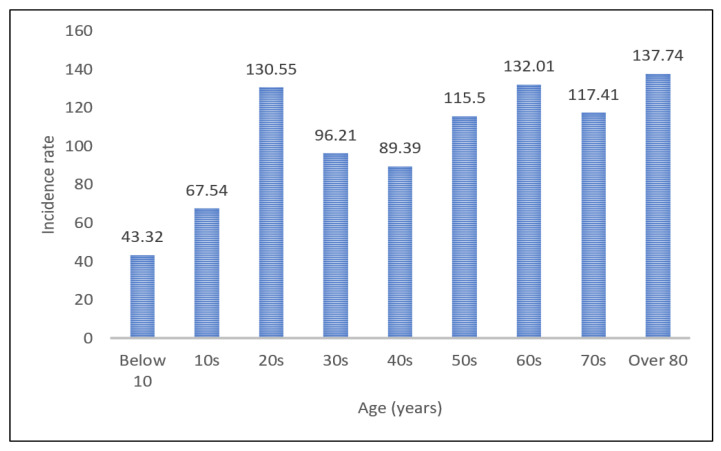
Incidence rate per 100,000 in South Korea as of 24 December 2020.

The number of deaths because of SARS-CoV-2 infection rises as the age of patients climbs. According to the Center for Disease Control and Prevention data, 80% of deaths from the coronavirus are associated with people over 65 years old [17]. Reports on the case fatality rate (CFR) in China and Italy found that the CFR for people aged under 60 was less than 2%, while the CFR for the aged over 60 years old was 20% [18]. In the United States, the CFR of those aged 65 or older was 3 to 27%, higher than that of younger people [19]. In Korea, the COVID-19-related CFR for the elderly was like other countries. Figure 3 shows the share of Korean coronavirus cases and CFR by age group. Over 70% of people infected with COVID-19 were aged under 50, whereas the CFR showed a rapid nonlinear rise for people aged over 60. Figure 4 shows the incidence rate of the coronavirus by age group. The number of confirmed cases per 100,000 population was relatively high among the elderly aged 60 or older.

**Figure 5:**
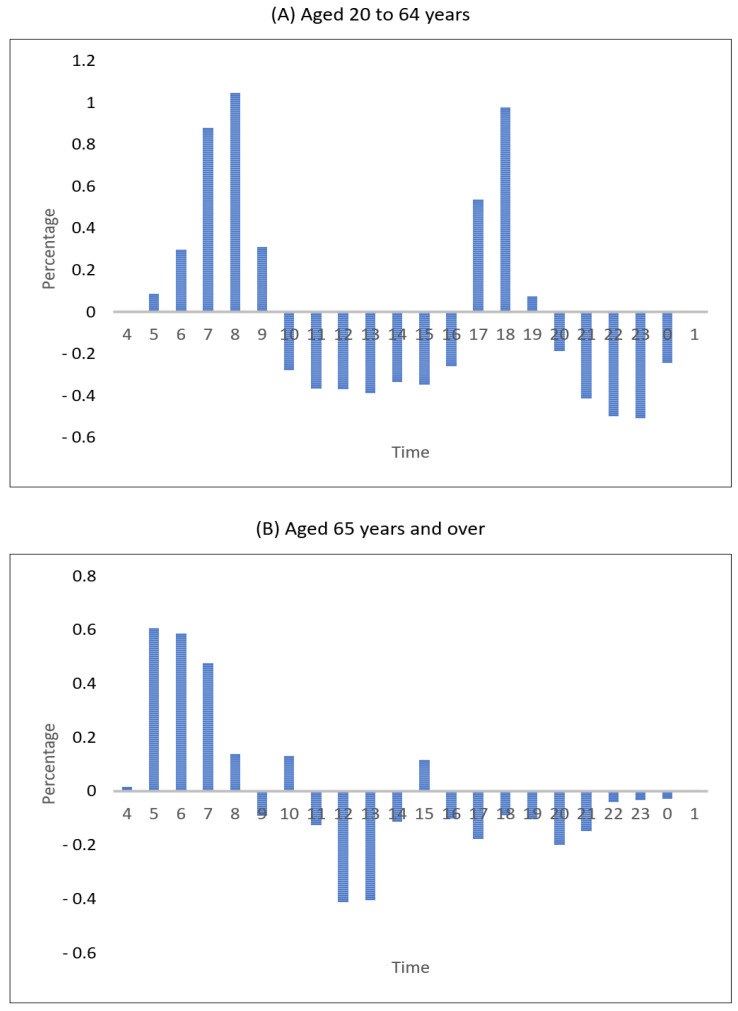
Change in the share of subway use hours.

The COVID-19 pandemic has led to changes in the hours of subway use and the number of rides. Figure 5 shows a change in the share of the number of subway rides by the hour in 2020 and 2019. Compared with 2019, the number of subway rides of passengers aged 20 to 64 in 2020 rose by 0.2% to 1% during 6–9 a.m. (commute to work) and rose by 0.5 to 0.9% during 5–6 p.m. (commute from work). In hours other than those, the use of the subway transit dropped. This was likely to be attributed to the reduction of operating hours of stores owing to the social distancing rule and a decrease in the number of permitted persons for meetings and dinners. The subway use for passengers aged 65 and over rose by 0.5% during 5–7 a.m., and the use of the subway decreased during hours other than this. Such changes in time for the use of the subway for the aged people who were not constrained by time for their social activities seemed to be meant to reduce human contact and restrict external activities voluntarily to avoid SARS-CoV-2 infection.

## 2. Materials and Methods

### 2.1. Data

This study collected data from the Seoul Bigdata Campus and the Seoul Open Data Plaza of the Seoul Metropolitan government. The period for this analysis ran from January to September in 2018, 2019, and 2020. Data for the cases of COVID-19 in Seoul relied on data from the Seoul Metropolitan government and the data for the Korean cases were from the Center for Systems Science and Engineering of Johns Hopkins University.

#### 2.1.1. Seoul Bigdata Campus

**Figure 6:**
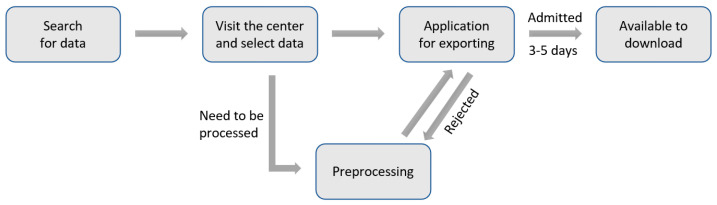
Data collection procedure of the Seoul Bigdata Campus.

The Seoul Bigdata Campus collects big data provided by organizations of the Seoul Metropolitan government and provides it to public institutions, academics, and private companies to help with research and solve social issues. As the collected data contains personal information, the sources of that data needed to be visited to get the preprocessing and approval for exporting that data before it can be used. Figure 6 shows the procedure of using raw data obtained from the Seoul Bigdata Campus.

The data on subway use in Seoul was the raw data provided by Tmoney Co., Ltd. to the Seoul Bigdata Campus, which could not be used without preprocessing and approval for exporting it. In Seoul, passengers must use smart cards equipped with a transportation card function to use the subway, and thus data from Tmoney Co., Ltd. that lists the details of the transportation card transactions are highly reliable. This data includes the date and time for the subway use, the station for departure and arrival, the type of subway user (type of subway user refers to senior citizens, persons of national merit, children, foreign senior citizens, general citizens, disabled persons, and youth; about 80% of subway users are general citizens aged between 20 and 64, the elderly aged 65 and older account for 12 to 13% of users, and the rest account for 6 to 7%), and the number of passengers.

This research focused on the changes in demand for the subway from residents in Seoul. While Seoul Metro also covers subway stations in both Seoul Metropolitan City and Gyeonggi-do province, this study conducted analysis only for subway stations located in 25 districts of Seoul Metropolitan City and collected data of the Korea Smart Card Co. on the number of rides by the district at departure stations.

#### 2.1.2. Seoul Open Data Plaza

Like the Seoul Bigdata Campus, Seoul Open Data Plaza collects data provided by organizations of the Seoul Metropolitan government. However, unlike Seoul Bigdata Campus, it makes public data open to all citizens and does not require visits to organizations providing such data. Seoul Open Data Plaza provides links to providers of a vast amount of data such that the latest data can be obtained from the links. Data for independent variables used in this study were based on the data of Seoul Open Data Plaza, the Ministry of Land, Infrastructure, and Transport, and the Transport Operation and Information Service of Seoul.

### 2.2. Empirical Model

This study used panel data of 25 districts in Seoul. Analyses were conducted in Stata/SE (version 16.1, StataCorp., College Station, TX, USA). Using the fixed-effects model, changes in the pattern of subway use by passengers aged 20 to 64 and passengers aged 65 and older amid the COVID-19 pandemic were analyzed. Fixed-effects models are effective at controlling for omitted variable bias because of unobserved heterogeneity [20]. Hence, this kind of model was suitable for controlling the environmental characteristics of each district of Seoul Metropolitan City.

The Korean government adjusted the social distancing levels based on variations of the number of confirmed cases. In phase 2 or higher of social distancing, there were significantly more restrictions on the economically active population than below phase 2. Hence, it was necessary to distinguish the period according to the social distancing level. The formula was as follows:

(1) $$y_{it}=\beta_{1}\mathrm{SDLv}1+\beta_{2}\mathrm{SDLv}2+\beta_{3}{\mathrm{CarSpeed}}_{it}+\beta_{4}{\mathrm{Pop}}_{it}+\beta_{5}{\mathrm{OwnCar}}_{it}+\beta_{6}{\mathrm{Wealth}}_{it}+\alpha_{i}+\varepsilon_{it}.$$

In Formula (1) above, subscript *i* means the districts of Seoul and *t* refers to the monthly data. $y_{it}$ is the logarithm of the number of subway rides per month in each district. SDLv1 is a dummy variable taking 1 for January, February, May, June, and July 2020 when social distancing was in phase 1 to 1.5 and taking 0 for other periods. SDLv2 is a dummy variable taking 1 for March, April, August, and September of 2020 when social distancing was at phase 2 or higher and taking 0 for other periods. CarSpeed is the average speed of automobiles measured by each district. Pop refers to the population registered in each district office. OwnCar refers to the share of privately owned cars out of the total registered vehicles in each district. Wealth is the average price per square meter of an apartment house in each district. $\alpha_{i}$ refers to the time-invariant location fixed effects. $\beta_{i}$ and $\varepsilon_{it}$ are coefficients and error term respectively.

Next, the elasticity of subway use in response to the number of COVID-19 cases was measured. The elasticity indicates the sensitivity of the number of subway uses in response to an increase in cases, and also reveals the differing sensitivity to the number of cases among age groups. The formula was as follows:

(2) $$y_{it}=\beta_{1}{\mathrm{Covid}}_{t}+\beta_{2}{\mathrm{CarSpeed}}_{it}+\beta_{3}{\mathrm{Pop}}_{it}+\beta_{4}{\mathrm{OwnCar}}_{it}+\beta_{5}{\mathrm{Wealth}}_{it}+\alpha_{i}+\varepsilon_{it}.$$

Additionally, concerning the Covid variable, the number of cases in Seoul and the number of cases in Korea were used to see the differences between residents’ responses to infection in local communities and nationwide, respectively.

Last, if the decrease in mass transit demand was due to the perception of the risk of the infection from the coronavirus, regions with a higher subway demand would experience a larger decrease in subway use. Areas with a large number of subway stations and transfer stations have a higher demand for subway use than other areas due to better accessibility to subways. The high demand for subway use not only increases the population density but also increases the floating population. This environment makes it easier for people to be more exposed to SARS-CoV-2 infection. Accordingly, analysis by period was made separately for areas with 16 or more stations, including transfer stations, and areas with less than 16 stations.

## 3. Results

**Figure 7:**
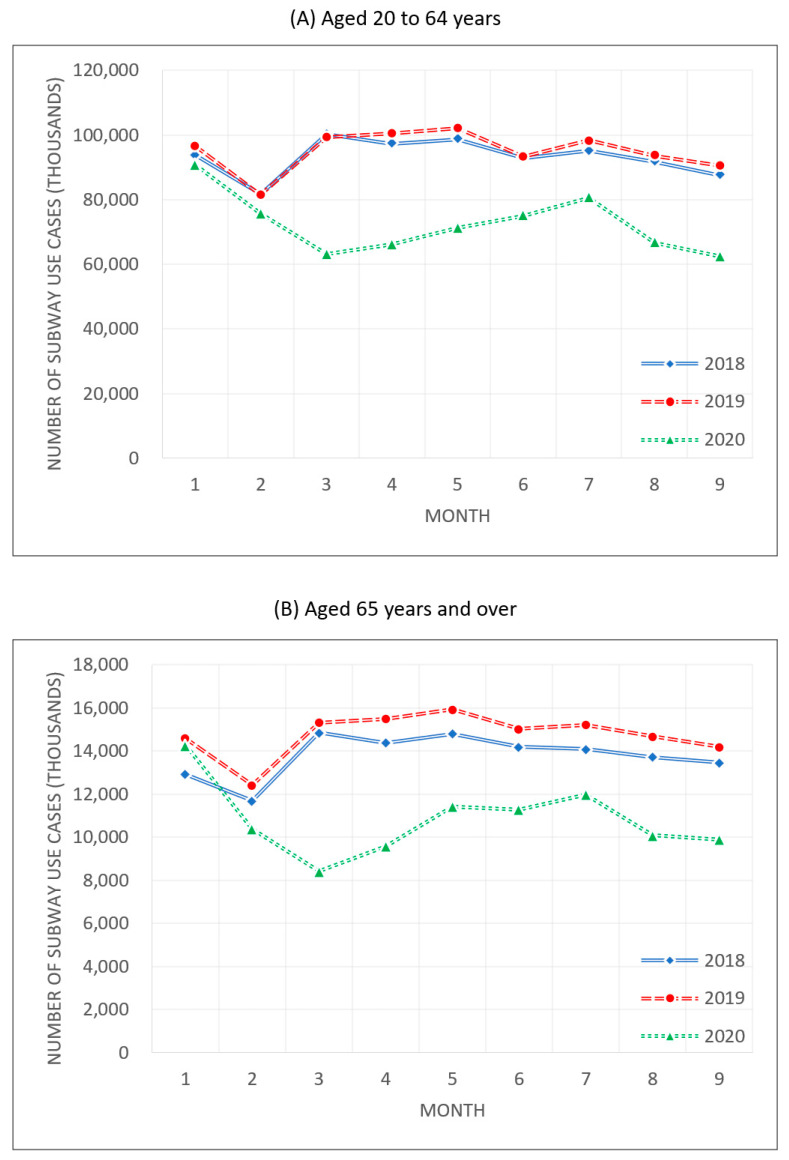
Number of subway uses during 2018–2020.

The Seoul Metropolitan Subway with a potentially higher rate of SARS-CoV-2 cluster infections saw the number of users declining significantly since the outbreak of the pandemic. Figure 7 shows the changes in the number of rides for passengers aged between 20 and 64 and those aged 65 and older. During 2018 and 2019 before the pandemic, those aged between 20 and 64 took 94,130,730 subway rides a month on average, and those aged 65 and older took 14,278,200 rides per month. After the COVID-19 outbreak, people between 20 and 64 used the subway 72,398,975 times a month while those aged 65 and older used the subway 10,815,514 times a month, showing a dramatic decrease.

Table 1 shows the change in the number of subway rides in each age group. Under stronger social distancing levels, the subway use of the two groups decreased. People aged 20 to 64 decreased their number of subway rides by 13% in phases 1 and 1.5 of social distancing, and by 30% as the social distancing level rose to phase 2 or higher. Likewise, the elderly aged 65 and older reduced their subway use by 19% in phases 1 and 1.5 and by 42% in phase 2 or higher. This suggests changes in the users’ behavioral patterns to avoid cluster infection risk by reducing subway use during the periods when the number of cases of the coronavirus increased, and thus the social distancing level was heightened.

Although the subway use demand decreased in both the 20–64 years and 65+ years age groups, the subway use of the elderly fell more prominently regardless of the social distancing phase. For the economically active population of Seoul, the subway is an essential transportation means to commute to work. Accordingly, this study expected that in phase 1 of social distancing, the decrease in subway use demand by people aged 20 to 64 years would be smaller than that for the elderly aged 65 years and older, and this expectation was consistent with analysis results. When the social distancing level was lifted to phase 2 or higher, the subway use demand by the economically active population decreased by a larger margin than for people aged 65 years and older due to activity-related reasons, such as remote work and a ban on nonessential meetings and events. Nevertheless, in phase 2 or higher of social distancing, subway use demand by people aged 65 years and older dropped by a significantly larger margin than the economically active population. For the elderly, the strengthening of social distancing did not change much in daily life, but the increase in the possibility of SARS-CoV-2 infection caused decision-making changes. Therefore, the result implies that the subway use demand by people aged 65 years and older was affected more by the voluntary restraint of mobility to avoid coronavirus infection than the social distancing policy.

Table 2 shows the change in subway use in tandem with the increase in the number of coronavirus cases. Both in Seoul and Korea as a whole, the increase in the number of coronavirus cases was negatively related to subway use. However, subway use was more sensitive to the number of cases in Seoul than to the number of cases nationwide. For subway users aged 20 to 64 years, a 1% increase in the number of Seoul cases led to a decrease in subway use by 0.06%, but a 1% increase in the number of cases nationwide reduced subway use by 0.03%. As for the elderly aged 65 years and older, the elasticity of subway use was higher in response to the number of cases in both Seoul and the whole nation than for people aged 20 to 64 years. A 1% increase in the number of Seoul cases reduced subway use by 0.08%, while a 1% increase in the number cases nationwide led to a decrease in subway rides by 0.06%. This finding suggests that despite the differing elasticities of subway use in response to the number of cases in a given region and the nation as a whole, the sensitivity to the risk of SARS-CoV-2 infection was higher for the aged people than for the younger people.

The results of the analysis given above confirmed that subway use demand decreased because of the coronavirus and that the aged were more sensitive to the risks associated with the virus than other age groups. Table 3 illustrates the analysis results by area based on the number of subway stations, including transfer stations. The findings revealed that the change in subway use pattern to avoid the infection risk was similar in all areas. Passengers aged 65 years and older decreased their number of subway rides more than those aged 20 to 64 years, regardless of the number of subway stations. Moreover, the elderly reduced their number of subway rides by a larger margin in areas with 16 or more stations, including transfer stations, than in areas with less than 16 stations. Both groups showed a greater drop in mass transit demand in areas with a large number of subway stations. This implies that people were trying to avoid high-risk areas.

## 4. Discussion

The results of this study showed the change in subway use demand in Seoul after the outbreak of SARS-CoV-2. The significant decrease in mass transit demand in both groups suggested that people’s mobility was greatly affected by the risk of infection. In particular, people aged 65 years and over avoided using the subway more than those aged between 20 and 64 years. The sensitivity to the risk of SARS-CoV-2 infection was also found to be high in the elderly. This suggests that because of the high fatality rate from the coronavirus among the elderly, the elderly’s fear of the pandemic was stronger, leading to an avoidance of public transportation. In sum, this study suggests that the differences in perceptions of the risk of coronavirus varied with age, and this was manifested through their mobility changes. The decline in people’s mobility cannot be interpreted as just an evasive behavior due to the risk of coronavirus infection. The decrease in spaces of consumption can be one factor that reduces mobility. These include a meeting cancellation, store closures, prohibition of leisure activities, and so on. The deteriorating labor market conditions can also contribute to the change of mobility. A decrease in income due to unemployment would shrink the amount of consumption, which can lead to a decline in mobility. There can be many other factors that can cause changes in human mobility. In this study, we focused on the overall change in people’s mobility by age. Therefore, if the socioeconomic factors mentioned above can be analyzed with mobility changes, we believe that the effect of infection risk on mobility reduction can be identified in detail. Furthermore, through an analysis of the relationship between mobility and the regional economic damage due to the coronavirus, we will be able to find the link between the consumption pattern and human mobility.

## 5. Conclusions

As the COVID-19 pandemic is protracted for an extended period, cases of COVID-19 are found in all age groups, but a high case fatality rate is still concentrated in the elderly. This means that in older adults, a coronavirus infection is more likely to result in the loss of life. In this respect, the difference in the human mobility between the young and the elderly presented in this study can be said to be a natural result. However, human mobility is associated with socioeconomic activities; therefore, it is difficult to explain the change in mobility solely in terms of the coronavirus risk. In order to analyze the effects of the coronavirus in more detail, an analysis of factors by age will be needed.

The Korean government is implementing a subsidy policy and plans to give additional subsidies to help small business owners who are suffering due to the COVID-19 pandemic. Since not all small business owners have suffered the same damage, the controversy over the target of the COVID-19 subsidy is increasing. The biggest impact for small business owners is the decline in sales due to people’s reduced mobility. Mobility encompasses various types of movement for living, leisure, consumption, and production. Therefore, if there is a regional analysis using the big data based on the result of mobility changes of this study, it will be helpful for the government to decide upon a subsidy policy.

## Figures and Tables

**Table 1:** Panel results: change in the subway demand by age and period.

Independent Variable	Dependent Variable	
	Log(Number of Subway Use Cases)	
	Aged 20 to 64 Years Old	Over 65 Years Old
Social distancing level 1 period	−0.1301 ***	−0.1973 ***
	(0.0143)	(0.0109)
Social distancing level 2 period	−0.3092 ***	−0.4212 ***
	(0.0208)	(0.0158)
Average car speed	−0.0309 ***	−0.0437 ***
	(0.0061)	(0.0045)
Population	0.0016	−0.0009
	(0.0011)	(0.0013)
Percentage of cars that were privately owned	−0.0314 **	−0.0006
	(0.0146)	(0.0079)
Average apartment price per square meter	−0.0001 *	0.0002 ***
	(0.0000)	(0.0000)
Cons	18.0675 ***	14.3751 ***
	(1.3860)	(0.7550)
R-squared	0.7674	0.7782
Observations	675	675
Location Fixed Effect	Yes	Yes

Note: Cluster robust standard errors are given in parenthesis. * *p* < 0.1, ** *p* < 0.05, *** *p* < 0.01.

**Table 2:** Panel results: elasticity of subway use demand by age in response to the number of COVID-19 cases.

Independent Variable	Dependent Variable			
	Log (the Number of Subway Use Cases)			
	Aged 20 to 64 Years Old		Over 65 Years Old	
Log(number of cases in Seoul)	−0.0600 ***	-	−0.0810 ***	-
	(0.0051)		(0.0048)	
Log(number of cases in Korea)	-	−0.0357 ***	-	−0.0627 ***
		(0.0022)		(0.0022)
Average car speed	−0.0252 ***	−0.0315 ***	−0.0501 ***	−0.0410 ***
	(0.0044)	(0.0046)	(0.0034)	(0.0029)
Population	−0.0002	0.0016	−0.0057 *	−0.0028
	(0.0029)	(0.0020)	(0.0033)	(0.0020)
Percentage of cars that were privately owned	0.0284	−0.0323	0.1656 ***	0.0871 **
	(0.0344)	(0.0430)	(0.0395)	(0.0410)
Average apartment price per squre meter	0.0001	−0.0008 ***	0.0008 ***	−0.0000
	(0.0001)	(0.0001)	(0.0002)	(0.0001)
Cons	12.8778 ***	18.9296 ***	0.4762	7.2712 *
	(3.1716)	(3.7799)	(3.4244)	(3.5412)
R-squared	0.5893	0.6058	0.5671	0.7323
Observations	225	225	225	225
Location Fixed Effect	Yes	Yes	Yes	Yes

Note: Cluster robust standard errors are given in parenthesis. * *p* < 0.1, ** *p* < 0.05, *** *p* < 0.01.

**Table 3:** Panel results: change in the subway use demand with respect to the number of subway stations.

Independent Variable	Dependent Variable			
	Log (Number of Subway Use Cases)			
	Less than 16 Stations		At Least 16 Stations	
	Aged 20–64	Over 65	Aged 20–64	Over 65
Social distancing level 1	−0.1211 ***	−0.1751 ***	−0.1462 ***	−0.2193 ***
	(0.0185)	(0.0109)	(0.0155)	(0.0146)
Social distancing level 2	−0.2928 ***	−0.3877 ***	−0.3349 ***	−0.4538 ***
	(0.0301)	(0.0164)	(0.0227)	(0.0197)
Average car speed	−0.0287 ***	−0.0500 ***	−0.0304 ***	−0.0386 ***
	(0.0069)	(0.0081)	(0.0085)	(0.0059)
Population	0.0009	−0.0017 **	0.0016	−0.0006
	(0.0015)	(0.0007)	(0.0020)	(0.0032)
Percentage of cars that were privately owned	0.0335	−0.0072	−0.0462 ***	−0.0021
	(0.0358)	(0.0223)	(0.0131)	(0.0112)
Average apartment price per squre meter	−0.0002 **	0.0001 **	−0.0000	0.0002 ***
	(0.0000)	(0.0000)	(0.0000)	(0.0000)
Cons	11.9597 ***	15.2013 ***	19.7357 ***	14.4994 ***
	(3.0686)	(2.0075)	(1.3862)	(1.1523)
R-squared	0.7617	0.7740	0.7833	0.7861
Observations	351	351	324	324
Location Fixed Effect	Yes	Yes	Yes	Yes

Note: Cluster robust standard errors are given in parenthesis. ** *p* < 0.05, *** *p* < 0.01.

## Data Availability

The data presented in this study are available on request from the corresponding author. The data are not publicly available due to the institutional data policy.

## Appendix A

**Table A1:** Statistics on mass transit use in Seoul during 2018 to 2020.

Subway Use (Number of Rides)					
Age Group	Year	Mean	Min	Max	SD
Total	2018	114,920,221	100,453,195	123,690,832	6,874,797
	2019	117,422,892	100,588,781	126,864,286	7,447,456
	2020	87,755,394	75,018,658	111,563,274	11,061,701
20 to 64 years	2018	93,219,289	81,810,105	100,149,309	5,413,155
	2019	95,042,169	81,525,891	102,024,162	5,930,896
	2020	72,398,975	62,409,113	90,678,007	8,662,742
Over 65 years	2018	13,789,837	11,678,379	14,856,445	944,777
	2019	14,766,562	12,435,456	15,931,192	960,168
	2020	10,815,514	8,410,424	14,265,611	1,587,980
**Bus Use (Number of Rides)**					
Total	2018	138,688,913	120,557,605	149,004,732	8,411,540
	2019	144,219,373	120,915,659	154,087,859	9,389,018
	2020	113,406,948	98,244,352	134,661,069	11,473,515
